# (*E*)-2-{4-[(Pyridin-2-yl)methyl­idene­amino]­phen­yl}acetic acid

**DOI:** 10.1107/S1600536812006472

**Published:** 2012-02-17

**Authors:** Zhen Nu Zheng, Soon W. Lee

**Affiliations:** a Department of Chemistry (BK21), Sungkyunkwan University, Natural Science Campus, Suwon 440-746, Republic of Korea

## Abstract

The title mol­ecule, C_14_H_12_N_2_O_2_, forms a dimeric unit linked by a pair of symmetry-equivalent O—H⋯N hydrogen bonds. The aromatic rings are significantly twisted from each other with a dihedral angle of 44.04 (4)°.

## Related literature

For transition-metal or lanthanide coordination polymers containing linking ligands related to the title mol­ecule, see: Han & Lee (2012 ▶); Jang & Lee (2010 ▶); Li *et al.* (2011 ▶); Yun *et al.* (2009 ▶); Zhang *et al.* (2004 ▶). For *d*–*f* metal–organic frameworks based on pyrid­yl–carboxyl­ate-type linking ligands, see: Chen *et al.* (2011 ▶, 2010 ▶); Tang *et al.* (2010 ▶); Yue *et al.* (2011 ▶); Zhu *et al.* (2010 ▶).
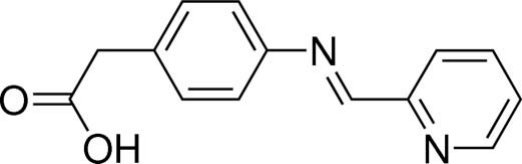


## Experimental

### 

#### Crystal data

C_14_H_12_N_2_O_2_
*M* *_r_* = 240.26
Monoclinic, 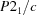

*a* = 4.1558 (1) Å
*b* = 25.7790 (5) Å
*c* = 11.2213 (2) Å
β = 96.623 (1)°
*V* = 1194.14 (4) Å^3^
*Z* = 4
Mo *K*α radiation
μ = 0.09 mm^−1^
*T* = 296 K
0.32 × 0.28 × 0.22 mm

#### Data collection

Bruker APEXII CCD diffractometer
Absorption correction: multi-scan (*SADABS*; Sheldrick, 1996 ▶) *T*_min_ = 0.971, *T*_max_ = 0.980
19023 measured reflections
2999 independent reflections
2113 reflections with *I* > 2σ(*I*)
*R*_int_ = 0.048

#### Refinement

*R*[*F* ^2^ > 2σ(*F* ^2^)] = 0.042
*wR*(*F* ^2^) = 0.120
*S* = 1.05
2999 reflections
167 parameters
H atoms treated by a mixture of independent and constrained refinement
Δρ_max_ = 0.24 e Å^−3^
Δρ_min_ = −0.19 e Å^−3^

### 

Data collection: *APEX2* (Bruker, 2007 ▶); cell refinement: *SAINT* (Bruker, 2007 ▶); data reduction: *SAINT*; program(s) used to solve structure: *SHELXS97* (Sheldrick, 2008 ▶); program(s) used to refine structure: *SHELXL97* (Sheldrick, 2008 ▶); molecular graphics: *SHELXTL* (Bruker, 2007 ▶); software used to prepare material for publication: *SHELXTL*.

## Supplementary Material

Crystal structure: contains datablock(s) I, global. DOI: 10.1107/S1600536812006472/fy2044sup1.cif

Structure factors: contains datablock(s) I. DOI: 10.1107/S1600536812006472/fy2044Isup2.hkl

Supplementary material file. DOI: 10.1107/S1600536812006472/fy2044Isup3.cml

Additional supplementary materials:  crystallographic information; 3D view; checkCIF report

## Figures and Tables

**Table 1:** Hydrogen-bond geometry (Å, °)

*D*—H⋯*A*	*D*—H	H⋯*A*	*D*⋯*A*	*D*—H⋯*A*
O1—HO1⋯N1^i^	0.95 (2)	1.73 (2)	2.6686 (15)	165.9 (18)

Symmetry code: (i) 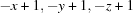.

**Fig. 1.**
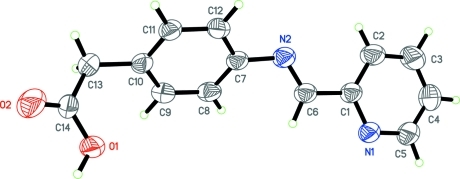
The molecular structure of the title compound showing the atomic numbering and 50% probability displacement ellipsoids.

**Fig. 2.**
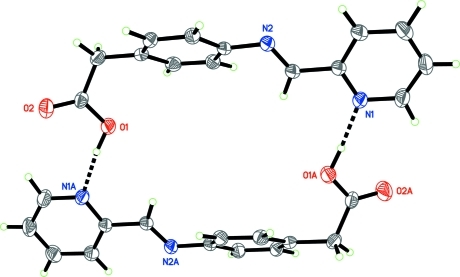
A dimeric unit formed by the O–H···N hydrogen bonds. Suffix A in atom labels indicates the symmetry operation –x+1, –y+1, –z+1.

## supplementary crystallographic information

### Comment

Coordination polymers are typically prepared by employing linking ligands possessing various terminal groups, including pyridyl–pyridyl, pyridyl–sulfonate, pyridyl–amine, carboxylate–carboxylate, and pyridyl–carboxylate terminals (Han & Lee, 2012; Jang & Lee, 2010; Li *et al.*, 2011; Yun *et al.*, 2009; Zhang *et al.*, 2004). Almost all known polymers contain either *d*- or *f*-block metals. On the other hand, several pyridyl–carboxylate type ligands were recently utilized for the preparation of coordination polymers containing both *d*- and *f*-block metals within their frameworks (Chen *et al.*, 2011; Chen *et al.*, 2010; Tang *et al.*, 2010; Yue *et al.*, 2011; Zhu *et al.*, 2010). In our ongoing study of coordination polymers, a new potential linking ligand with the pyridyl–carboxylate terminals was synthesized. We herein report its crystal structure.

The molecular structure of the title molecule with the atom-labeling scheme is given in Figure 1, which clearly shows both the pyridyl and the carboxylate terminals. The π-conjugation system of the entire molecule is interrupted due to the CH_2_ fragment in the terminal CH_2_COOH group. Two planar 6-membered rings (2-pyridyl and phenyl rings) are significantly twisted from each other with the dihedral angle of 44.04 (4)°. The torsion angle of C1–C6–N2–C7 is 175.9 (1)°. The N2–C6 bond length [1.265 (2) Å] clearly indicates a C=N double bond. The N1···O1 and N1···O2 separations are 8.457 (1) and 10.249 (2) Å, respectively. As shown in Figure 2, two molecules are connected by the strong intermolecular hydrogen bonds of the O–H···N type (Table 1).

### Experimental

At room temperature, 4-(aminophenyl)acetic acid (1.00 g, 6.6 mmol) was dissolved in hot methanol (30 ml), and then 2-pyridinecarboxaldehyde (0.71 g, 6.6 mmol) was added slowly. The mixture was refluxed at 65 °C for 2 h. After being slowly air-cooled, the resulting solution was filtered and concentrated to one-fourth of its original volume with a rotary evaporator, and then allowed to stand for 24 h. The resulting green crystals were separated by filtration, washed with methanol (10 ml × 3), and then vacuum-dried to give the title compound (1.34 g, 5.6 mmol, 85.0% yield). mp: 411–413 K. ^1^H NMR (500 MHz, CD_3_SOCD_3_, δ): 8.70–8.71 (m, 1H, pyridine N–C*H*), 8.58 (s, 1H, N=C*H*), 8.14 (d, 1H, aromatic proton), 7.91–7.95 (m, 1H, aromatic proton), 7.49–7.52 (m, 1H, aromatic proton), 7.27–7.33 (m, 4H, aromatic protons), 3.60 (s, 2H, C*H*_2_). ^13^C{^1^H} NMR (125 MHz, CD_3_SOCD_3_, δ): 173.3, 161.1, 154.8, 150.3, 149.6, 137.6, 134.5, 130.9, 126.3, 122.4, 121.9, 40.5. IR (KBr, cm^-1^): 3057 (w), 2885 (w), 2826 (w), 2109 (w), 2014 (w), 1925(w), 1735 (*m*), 1635 (*s*), 1508 (*s*), 1461 (*m*), 1385 (w), 1142 (*m*), 1023 (*m*), 856 (*m*), 803 (w), 776 (w), 724 (w), 612 (w).

### Refinement

All non-hydrogen atoms were refined anisotropically. C-bound H atoms were positioned geometrically [C—H = 0.93–0.97 Å] and allowed to ride on their parent atoms, with *U*_iso_(H) = 1.2*U*_eq_(C). Atom HO1 was located in a difference Fourier map and refined isotropically.

### Crystal data

**Table:**

C_14_H_12_N_2_O_2_	*F*(000) = 504
*M**_r_* = 240.26	*D*_x_ = 1.336 Mg m^−^^3^
Monoclinic, *P*2_1_/*c*	Mo *K*α radiation, λ = 0.71073 Å
Hall symbol: -P 2ybc	Cell parameters from 6688 reflections
*a* = 4.1558 (1) Å	θ = 2.4–27.7°
*b* = 25.7790 (5) Å	µ = 0.09 mm^−^^1^
*c* = 11.2213 (2) Å	*T* = 296 K
β = 96.623 (1)°	Block, green
*V* = 1194.14 (4) Å^3^	0.32 × 0.28 × 0.22 mm
*Z* = 4	

### Data collection

**Table:**

Bruker APEXII CCD diffractometer	2999 independent reflections
Radiation source: sealed tube	2113 reflections with *I* > 2σ(*I*)
Graphite monochromator	*R*_int_ = 0.048
φ and ω scans	θ_max_ = 28.4°, θ_min_ = 1.6°
Absorption correction: multi-scan (*SADABS*; Sheldrick, 1996)	*h* = −5→5
*T*_min_ = 0.971, *T*_max_ = 0.980	*k* = −34→34
19023 measured reflections	*l* = −14→14

### Refinement

**Table:**

Refinement on *F*^2^	Primary atom site location: structure-invariant direct methods
Least-squares matrix: full	Secondary atom site location: difference Fourier map
*R*[*F*^2^ > 2σ(*F*^2^)] = 0.042	Hydrogen site location: inferred from neighbouring sites
*wR*(*F*^2^) = 0.120	H atoms treated by a mixture of independent and constrained refinement
*S* = 1.05	*w* = 1/[σ^2^(*F*_o_^2^) + (0.0594*P*)^2^ + 0.0884*P*] where *P* = (*F*_o_^2^ + 2*F*_c_^2^)/3
2999 reflections	(Δ/σ)_max_ < 0.001
167 parameters	Δρ_max_ = 0.24 e Å^−^^3^
0 restraints	Δρ_min_ = −0.19 e Å^−^^3^

### Special details

**Table:**

Geometry. All e.s.d.'s (except the e.s.d. in the dihedral angle between two l.s. planes) are estimated using the full covariance matrix. The cell e.s.d.'s are taken into account individually in the estimation of e.s.d.'s in distances, angles and torsion angles; correlations between e.s.d.'s in cell parameters are only used when they are defined by crystal symmetry. An approximate (isotropic) treatment of cell e.s.d.'s is used for estimating e.s.d.'s involving l.s. planes.
Refinement. Refinement of *F*^2^ against ALL reflections. The weighted *R*-factor *wR* and goodness of fit *S* are based on *F*^2^, conventional *R*-factors *R* are based on *F*, with *F* set to zero for negative *F*^2^. The threshold expression of *F*^2^ > σ(*F*^2^) is used only for calculating *R*-factors(gt) *etc*. and is not relevant to the choice of reflections for refinement. *R*-factors based on *F*^2^ are statistically about twice as large as those based on *F*, and *R*- factors based on ALL data will be even larger.

### Fractional atomic coordinates and isotropic or equivalent isotropic displacement parameters (Å^2^)

**Table:**

	*x*	*y*	*z*	*U*_iso_*/*U*_eq_	
O1	0.0141 (3)	0.59412 (4)	0.29429 (8)	0.0559 (3)	
O2	−0.1260 (4)	0.62479 (4)	0.11244 (10)	0.0854 (4)	
N1	0.6832 (3)	0.31705 (4)	0.64271 (10)	0.0498 (3)	
N2	0.2167 (3)	0.35524 (4)	0.37215 (9)	0.0468 (3)	
C1	0.5054 (3)	0.30923 (5)	0.53680 (11)	0.0433 (3)	
C2	0.4438 (4)	0.25998 (5)	0.49147 (14)	0.0582 (4)	
H2	0.3247	0.2554	0.4168	0.070*	
C3	0.5605 (4)	0.21775 (6)	0.55782 (16)	0.0686 (5)	
H3	0.5219	0.1843	0.5285	0.082*	
C4	0.7336 (4)	0.22551 (6)	0.66725 (15)	0.0671 (5)	
H4	0.8104	0.1975	0.7146	0.081*	
C5	0.7917 (4)	0.27542 (6)	0.70586 (13)	0.0626 (4)	
H5	0.9130	0.2806	0.7799	0.075*	
C6	0.3776 (3)	0.35647 (5)	0.47493 (11)	0.0439 (3)	
H6	0.4165	0.3884	0.5126	0.053*	
C7	0.0845 (3)	0.40250 (4)	0.32274 (11)	0.0405 (3)	
C8	−0.0533 (3)	0.43940 (5)	0.39151 (11)	0.0452 (3)	
H8	−0.0541	0.4341	0.4735	0.054*	
C9	−0.1887 (3)	0.48373 (5)	0.33884 (12)	0.0457 (3)	
H9	−0.2835	0.5078	0.3858	0.055*	
C10	−0.1870 (3)	0.49330 (4)	0.21741 (11)	0.0400 (3)	
C11	−0.0539 (3)	0.45583 (5)	0.14942 (11)	0.0433 (3)	
H11	−0.0518	0.4612	0.0676	0.052*	
C12	0.0758 (3)	0.41058 (5)	0.20048 (11)	0.0449 (3)	
H12	0.1575	0.3855	0.1526	0.054*	
C13	−0.3321 (3)	0.54233 (5)	0.16057 (13)	0.0491 (3)	
H13A	−0.3663	0.5372	0.0744	0.059*	
H13B	−0.5432	0.5474	0.1877	0.059*	
C14	−0.1385 (3)	0.59138 (5)	0.18526 (12)	0.0448 (3)	
HO1	0.130 (5)	0.6261 (9)	0.3040 (17)	0.097 (6)*	

### Atomic displacement parameters (Å^2^)

**Table:**

	*U*^11^	*U*^22^	*U*^33^	*U*^12^	*U*^13^	*U*^23^
O1	0.0796 (7)	0.0436 (5)	0.0408 (5)	−0.0069 (5)	−0.0092 (5)	0.0010 (4)
O2	0.1241 (11)	0.0643 (7)	0.0594 (7)	−0.0192 (7)	−0.0258 (7)	0.0201 (6)
N1	0.0604 (7)	0.0424 (6)	0.0431 (6)	0.0003 (5)	−0.0083 (5)	0.0016 (5)
N2	0.0582 (7)	0.0396 (5)	0.0403 (6)	−0.0062 (5)	−0.0044 (5)	0.0031 (4)
C1	0.0491 (7)	0.0402 (6)	0.0393 (7)	−0.0050 (5)	−0.0005 (5)	0.0026 (5)
C2	0.0734 (10)	0.0433 (7)	0.0538 (8)	−0.0101 (7)	−0.0099 (7)	−0.0004 (6)
C3	0.0904 (12)	0.0375 (7)	0.0746 (11)	−0.0061 (7)	−0.0038 (9)	0.0005 (7)
C4	0.0863 (12)	0.0442 (8)	0.0675 (10)	0.0086 (7)	−0.0052 (9)	0.0131 (7)
C5	0.0787 (11)	0.0537 (8)	0.0504 (8)	0.0069 (7)	−0.0145 (7)	0.0065 (6)
C6	0.0516 (7)	0.0380 (6)	0.0403 (7)	−0.0073 (5)	−0.0021 (6)	0.0010 (5)
C7	0.0477 (7)	0.0348 (6)	0.0370 (6)	−0.0092 (5)	−0.0043 (5)	0.0006 (5)
C8	0.0561 (8)	0.0460 (7)	0.0329 (6)	−0.0099 (6)	0.0021 (5)	−0.0003 (5)
C9	0.0499 (7)	0.0447 (7)	0.0426 (7)	−0.0035 (6)	0.0048 (6)	−0.0069 (5)
C10	0.0373 (6)	0.0386 (6)	0.0420 (7)	−0.0052 (5)	−0.0044 (5)	−0.0010 (5)
C11	0.0525 (7)	0.0438 (7)	0.0322 (6)	−0.0034 (5)	−0.0006 (5)	0.0014 (5)
C12	0.0569 (8)	0.0392 (6)	0.0378 (7)	−0.0013 (5)	0.0011 (6)	−0.0032 (5)
C13	0.0442 (7)	0.0492 (7)	0.0505 (8)	0.0048 (6)	−0.0090 (6)	−0.0010 (6)
C14	0.0495 (7)	0.0413 (7)	0.0420 (7)	0.0104 (5)	−0.0021 (6)	0.0019 (5)

### Geometric parameters (Å, º)

**Table:**

O1—C14	1.3135 (15)	C6—H6	0.9300
O1—HO1	0.95 (2)	C7—C12	1.3840 (17)
O2—C14	1.1925 (15)	C7—C8	1.3895 (18)
N1—C5	1.3353 (16)	C8—C9	1.3766 (17)
N1—C1	1.3404 (16)	C8—H8	0.9300
N2—C6	1.2651 (15)	C9—C10	1.3856 (18)
N2—C7	1.4222 (15)	C9—H9	0.9300
C1—C2	1.3806 (17)	C10—C11	1.3852 (18)
C1—C6	1.4703 (16)	C10—C13	1.5097 (17)
C2—C3	1.375 (2)	C11—C12	1.3813 (17)
C2—H2	0.9300	C11—H11	0.9300
C3—C4	1.364 (2)	C12—H12	0.9300
C3—H3	0.9300	C13—C14	1.5070 (19)
C4—C5	1.370 (2)	C13—H13A	0.9700
C4—H4	0.9300	C13—H13B	0.9700
C5—H5	0.9300		
			
C14—O1—HO1	109.6 (12)	C9—C8—C7	120.28 (11)
C5—N1—C1	117.88 (11)	C9—C8—H8	119.9
C6—N2—C7	118.28 (10)	C7—C8—H8	119.9
N1—C1—C2	121.65 (11)	C8—C9—C10	121.46 (12)
N1—C1—C6	115.24 (10)	C8—C9—H9	119.3
C2—C1—C6	123.11 (11)	C10—C9—H9	119.3
C3—C2—C1	119.33 (13)	C11—C10—C9	117.72 (11)
C3—C2—H2	120.3	C11—C10—C13	121.11 (11)
C1—C2—H2	120.3	C9—C10—C13	121.16 (12)
C4—C3—C2	119.17 (13)	C12—C11—C10	121.46 (11)
C4—C3—H3	120.4	C12—C11—H11	119.3
C2—C3—H3	120.4	C10—C11—H11	119.3
C3—C4—C5	118.54 (13)	C11—C12—C7	120.18 (12)
C3—C4—H4	120.7	C11—C12—H12	119.9
C5—C4—H4	120.7	C7—C12—H12	119.9
N1—C5—C4	123.39 (14)	C14—C13—C10	116.48 (10)
N1—C5—H5	118.3	C14—C13—H13A	108.2
C4—C5—H5	118.3	C10—C13—H13A	108.2
N2—C6—C1	122.27 (11)	C14—C13—H13B	108.2
N2—C6—H6	118.9	C10—C13—H13B	108.2
C1—C6—H6	118.9	H13A—C13—H13B	107.3
C12—C7—C8	118.82 (11)	O2—C14—O1	123.01 (13)
C12—C7—N2	118.70 (11)	O2—C14—C13	123.00 (12)
C8—C7—N2	122.37 (11)	O1—C14—C13	113.99 (11)

### Hydrogen-bond geometry (Å, º)

**Table:**

*D*—H···*A*	*D*—H	H···*A*	*D*···*A*	*D*—H···*A*
O1—H*O*1···N1^i^	0.95 (2)	1.73 (2)	2.6686 (15)	165.9 (18)

Symmetry code: (i) −*x*+1, −*y*+1, −*z*+1.
